# Participant experiences in a breastmilk biomonitoring study: A qualitative assessment

**DOI:** 10.1186/1476-069X-8-4

**Published:** 2009-02-18

**Authors:** Nerissa Wu, Michael D McClean, Phil Brown, Ann Aschengrau, Thomas F Webster

**Affiliations:** 1 Department of Environmental Health, Boston University School of Public Health, 715 Albany Street, Boston, MA 02118, USA; 2 Department of Sociology, Brown University, Box 1916, Providence, RI 02912, USA; 3 Department of Epidemiology, Boston University School of Public Health, 715 Albany Street, Boston, MA 02118, USA

## Abstract

**Background:**

Biomonitoring studies can provide information about individual and population-wide exposure. However they must be designed in a way that protects the rights and welfare of participants. This descriptive qualitative study was conducted as a follow-up to a breastmilk biomonitoring study. The primary objectives were to assess participants' experiences in the study, including the report-back of individual body burden results, and to determine if participation in the study negatively affected breastfeeding rates or duration.

**Methods:**

Participants of the Greater Boston PBDE Breastmilk Biomonitoring Study were contacted and asked about their experiences in the study: the impact of study recruitment materials on attitudes towards breastfeeding; if participants had wanted individual biomonitoring results; if the protocol by which individual results were distributed met participants' needs; and the impact of individual results on attitudes towards breastfeeding.

**Results:**

No participants reported reducing the duration of breastfeeding because of the biomonitoring study, but some responses suggested that breastmilk biomonitoring studies have the potential to raise anxieties about breastfeeding. Almost all participants wished to obtain individual results. Although several reported some concern about individual body burden, none reported reducing the duration of breastfeeding because of biomonitoring results. The study literature and report-back method were found to mitigate potential negative impacts.

**Conclusion:**

Biomonitoring study design, including clear communication about the benefits of breastfeeding and the manner in which individual results are distributed, can prevent negative impacts of biomonitoring on breastfeeding. Adoption of more specific standards for biomonitoring studies and continued study of risk communication issues related to biomonitoring will help protect participants from harm.

## Background

Biomonitoring, or the measure of chemicals that have accumulated in the body ("body burden"), has long been used to monitor individual and population-wide exposures to chemicals such as lead and mercury [1,2]. Epidemiologists and exposure assessors trying to understand exposure pathways between the environment and the individual, as well as advocacy groups drawing attention to the ubiquitous nature of environmental contaminants, have used biomonitoring as an effective tool [2,3].

Public interest in biomonitoring has increased; technology to measure chemicals in various media has also improved, resulting in more biomonitoring studies, identifying a wider range of chemicals in people. The ability to conduct biomonitoring has outpaced our knowledge about the ethics of biomonitoring [1]. As with any human subjects research, biomonitoring involves a number of ethical issues. Participants are often told that they have been exposed to and have a body burden of a chemical about which little is known. The risk to participants is therefore more of a psychological burden than the risk of physical harm. Examples of potential harm include the knowledge that exposure has taken place, the fear of a negative health outcome, and concern that chemicals may be passed to offspring. Another potential negative impact specific to breastmilk biomonitoring is the concern that participants will limit breastfeeding because of fears about the effect of toxins in breastmilk on infants [1].

### Impacts of Biomonitoring on Breastfeeding

There is significant evidence of the benefits of breastfeeding for the long- and short-term health of a child [4]. Accordingly, the American Academy of Pediatrics recommends that breastfeeding continue for the first year of life. There is very little published on the impact that learning about contaminants in breastmilk has on the decision to breastfeed. One study conducted following an industrial accident in Michigan examined the psychological effects of the subsequent contamination on nursing mothers in the community [5]. The 1973 incident involved the accidental introduction of polybrominated biphenyl (PBB) into animal feed which resulted in contamination of meat and dairy products which were subsequently distributed for human consumption [6]. Hatcher [5] interviewed 97 women who had obtained PBB analysis of their breastmilk. Fifteen percent chose to stop breastfeeding after receiving analytical results. A second study investigating exposures from the same accident measured the prevalence and duration of breastfeeding among 466 women exposed to PBB [7]. Women with high PBB levels were found to be no less likely to breastfeed than women with moderate or low levels. Duration of breastfeeding was not associated with PBB serum concentration, indicating that PBB did not negatively impact the ability to breastfeed. When asked why they chose to stop or limit breastfeeding, none of the women in the study cited concerns about PBB contamination [7].

More recently, there has been anecdotal evidence from lactation consultants that nursing mothers' concerns about contaminants in breastmilk increase after results of biomonitoring studies are publicized [8,9]. The National Research Council (NRC) Committee on Human Biomonitoring for Environmental Toxicants report, "Human Biomonitoring for Environmental Chemicals," acknowledged the potential for poorly designed breastmilk biomonitoring studies to negatively impact breastfeeding rates, yet asserted that studies should be conducted because of the importance of the data for efforts to reduce chemical body burdens [1].

### Communication of Biomonitoring Results

There is much debate about the issue of whether and how to provide individual results to the participants of research studies. The traditional clinical model does not include distribution of results unless they are clinically relevant [10,11]. However, many researchers support giving participants personal biomonitoring results, citing principles of respect and autonomy [10-12]. An expert panel convened to consider issues related to breastmilk biomonitoring affirmed that participants should have the option of obtaining individual results [13]. The NRC agrees that participants should have access to individual results, even if there is great uncertainty about the significance [1], and a lay panel convened at the Boston Consensus Conference on Biomonitoring felt similarly [14].

### Assessing the Impact of Breastmilk Biomonitoring Studies on Participants

Several expert panels have been convened to consider questions related to biomonitoring and to address issues such as participant recruitment and sampling, analytical issues, and methods for conducting studies without negatively impacting breastfeeding rates. [1,15-17].

Although the recommendations of the technical panels have been based on the collective experiences of researchers and experts, there has been little published on the perceptions of participants of biomonitoring studies to corroborate expert recommendations. Accordingly, we conducted the present study as a follow-up to the Greater Boston PBDE (GB-PBDE) biomonitoring study to better understand the impact of biomonitoring studies on breastfeeding women. The GB-PBDE biomonitoring study measured polybrominated diphenyl ethers (PBDEs) in the breastmilk of first-time mothers [18]. PBDEs are a group of chemicals used as flame retardants in many household products including electronics and furniture. Results of animal studies suggest reproductive and developmental effects, neurotoxicity, and endocrine disruption [19-21].

The primary objective of the follow-up study was to evaluate the impact of participating in a breastmilk biomonitoring study on breastfeeding practices; this included an assessment of how recruitment materials and other study literature were perceived by participants, whether or not participants wanted to obtain individual results, an evaluation of the manner in which results were communicated to participants, and determining if obtaining individual body burden results impacted attitudes towards breastfeeding.

## Methods

### Overview of the GB-PBDE Study

The Greater Boston PBDE (GB-PBDE) Body Burden Project was conducted during 2003–2005 [18]. The biomonitoring study was approved by the Boston University Medical Center Institutional Review Board (IRB) as was the subsequent follow-up study. Methodology for the GB-PBDE study has been published in detail elsewhere [18]. Recruitment was conducted at three different sites within the Greater Boston area including a health center in one ethnically diverse community (Lowell) and two private obstetrics/maternity centers in predominantly white, highly educated communities (Cambridge, Brookline). Biomonitoring participants were recruited in the late stages of pregnancy through the first post-partum weeks. Women who were eligible to participate in the study were given information on PBDEs and biomonitoring, information on the benefits of breastfeeding, and an informed consent form. Once consent was obtained, participants were asked a series of questions about general health, dietary and consumer habits, and household goods. Participants provided breastmilk samples two to eight weeks after giving birth, and a subset of participants provided a sample of household dust. Forty-six women participated in the study. Participant characteristics are summarized in Table 1.

**Table 1:** Participant characteristics for the GB-PBDE project and follow-up study

	GB-PBDE Project			Follow-Up Study		
	Lowell	Cambridge/Brookline	Total	Participants	Did Not Respond	Lost to Follow Up

**N**	5	41	46	31	8	7

**Age of Mother (years)**						
**Average**	21.0	33.6	32.2	33.5	31.0	27.7
**Range**	19–23	28–41	19–41	28–41	21–39	19–36
**Age of Baby at time of follow-up study (months)**						
**Average**				11.3	12.3	12.4
**Range**				(5.8–15)	(9–15)	(10–17)

**Smoking Status**						
**Current smoker**	40%	2.4%	6.5%	3%	0%	29%
**Former smoker**	0%	12.2%	10.9%	12.9%	12.5%	0%
**Never smoker**	60%	85.4%	82.6%	84.1%	87.5%	71%

**Highest Education Level Achieved**						
**HS graduate**	40%	0%	6.5%	0%	12.5%	29%
**Some college**	60%	0%	4.3%	0%	12.5%	14%
**College grad**	0%	100%	89%	100%	75%	57.1%

**Race/Ethnicity**						
**White**	40%	93%	87%	97%	63%	86%
**Asian**	60%	0%	7%	0%	25%	0%
**Latina**	0%	5%	4%	3%	0%	14%
**Af. American**	0%	2%	2%	0%	13%	0%

**At time of GB-PBDE Recruitment:**						
**Already breastfeeding**			63%	71%		
**Planning to breastfeed**			37%^a^	23%		
**Very likely/considering breastfeeding**				6%		

^a ^The follow-up questionnaire asked participants who were not already breastfeeding at the time they enrolled in the biomonitoring study to describe whether they were "planning to breastfeed", "very likely to breastfeed", or "considering breastfeeding." The original GB-PBDE questionnaire did not make this distinction; biomonitoring participants were described as either currently breastfeeding or planning to breastfeed.

The GB-PBDE study was designed to follow many of the recommendations set forth in the guidelines issued following the 2002 Technical Workshop on Human Milk Surveillance and Research on Environmental Chemicals in the United States: the need to recommend and support breastfeeding [15]; the importance of a thorough informed consent process; and the need to share results with participants [13]. The protocol was consistent with many of the recommendations issued at a subsequent Technical Workshop in 2005, such as 1) include protocols and information that emphasize that breastfeeding is the best infant nutrition; 2) include lactation consultants on study staff; 3) provide clearly written and non-alarming fact sheets; and 4) emphasize to participants that breastfeeding has been found to be beneficial for the long-term overall health of children even though environmental chemicals have historically been present in human milk [22]. Accordingly, while recruitment information clearly described the issue of bioaccumulation and concerns about PBDEs, communication with participants throughout the GB-PBDE study emphasized the benefits of breastfeeding, and study literature included a "breastfeeding is best" logo wherever possible. Recruitment materials included fact sheets on the health benefits of breastfeeding [23], contact information for breastfeeding resources, and information specifically addressing the issue of contaminants in breastmilk [24] (see Additional Files 1, 2, 3 for examples of materials distributed during participant recruitment). A lactation consultant was on-site for all recruitment efforts in Lowell and available by phone to all potential participants. A Community Advisory Board, including lactation consultants, environmental health educators, and community health workers reviewed all documents for content and readability.

Although the IRB initially objected to distribution of individual results, citing potential harm to participants, project staff felt that results could be made available in a way that provided support to participants and mitigated the risk of harm. Once individual biomonitoring data were available, a letter was sent inviting participants to call and speak to either the study coordinator or to a physician on our study team to obtain individual results. Alternatively, biomonitoring participants could attend a forum, open only to participants and their partners, at which results were communicated privately. The protocol was designed to fulfill participants' right to receive results, while not mandating that participants obtain individual data. Results were not mailed to participants because we felt that participants should have the opportunity to ask questions about the study immediately after receiving results.

Individual results were presented to participants in the context of the overall study, comparing individual results with average concentrations as well as the range. Participants were told that the study found PBDE body burden to be associated with consumption of dairy fat and meat as well as concentrations of PBDE in house dust, but that the study did not provide sufficient evidence to support specific recommendations to reduce body burden. Body burden concentrations were also compared to the concentrations given to animals in laboratory tests, and results of laboratory studies were discussed. However, participants were advised that human health implications for PBDE body burden were still unclear.

### Overview of the Follow-up Study

The follow-up study was conducted two months after biomonitoring results were made available, five to 17 months after biomonitoring participants had been initially recruited into the GB-PBDE study. Attempts were made to contact all 46 biomonitoring participants. The brief telephone questionnaire included fifteen questions covering several broad categories: the impact of learning about biomonitoring and PBDEs on attitudes towards breastfeeding; whether participants wanted to obtain individual body burden data; the impact of individual results on attitudes towards breastfeeding; and changes in environmental health awareness (see Additional File 4 for the follow-up questionnaire). Closed-ended questions were supplemented with open-ended follow-up questions and probes to more fully capture participants' attitudes and opinions regarding study design and communication of results.

### Data Analysis

Results of the follow-up questionnaire were transcribed by the interviewer. Responses to closed questions (often a yes/no answer) were summarized and analyzed quantitatively. Responses to open-ended probes were analyzed using coding techniques commonly utilized for qualitative research methods [25]. Participants' responses were examined, and recurrent themes in the responses were used to set up a framework through which to analyze results. This was an iterative method for which responses were reviewed for the development of codes and then re-read for the refinement and organization of the codes. Responses were first organized into broad categories, such as "no impact on breastfeeding duration" which closely followed the theme of the questionnaire. Codes within this category, such as "impacts of study literature" or "potential for impact with different results" were then devised based on the recurring themes in participants' responses.

## Results

Of the 46 participants of the GB-PBDE study, 31 (67%) were reached and agreed to participate in the follow-up study. Eight were left messages but did not call back; seven were lost to follow up. We were not able to reach any of the biomonitoring participants from Lowell (n = 5). As a result, non-participants and participants differed with regard to race and education. The majority of follow-up study participants were white and highly educated. Overall there appeared to be little difference between participants and non-participants with regard to maternal or baby age at the time of follow-up.

Of the 31 participants of the follow-up study, 22 (71%) had just given birth and had already initiated breastfeeding at the time they were recruited for the biomonitoring study. Nine (29%) were still pregnant at the time of initial recruitment; of these, seven (23%) were "definitely planning" to breastfeed while two (6%) were "very likely" or "considering" breastfeeding at that time. This is representative of the overall GB-PBDE study population; most participants were committed to or already breastfeeding at the time that they were recruited. Characteristics of participants and non-participants of the follow-up study are summarized in Table 1.

### Impacts on Attitudes towards Breastfeeding

Women were asked, "Did the information given to you about the study change how you felt about breastfeeding?" None of the 31 women reported having changed her plans to breastfeed because of the general information on biomonitoring or PBDEs, but four women reported that the study information made them think differently about breastfeeding. One woman responded:

It made me think that my surroundings were more toxic than I thought. There's some sorrow in learning about it, but I don't want to change things without knowing more. I just do the best I can... eating organic, breastfeeding.

Of these four women, two specifically identified the "breastfeeding is best" message that was highlighted throughout study literature as having a positive effect. As one woman articulated, "The initial information gave me a moment of hesitation but the literature was reassuring."

Four other respondents reported that the information given out reinforced their decision to breastfeed, citing "a renewed belief" in her decision, "another good reason to breastfeed" and "validation" of her decision. As one stated:

I had heard a lot of questions about breastfeeding – if it's still good to do because of all of the chemicals in our bodies. I was glad to hear that my opinion was correct.

### Individual Biomonitoring Results

Individual results were made available to participants as part of the biomonitoring study, two months before the follow-up questionnaire was conducted. Letters were sent to all 46 GB-PBDE study participants informing them that biomonitoring results were available. One letter was returned as undeliverable. Fourteen of the participants (30%) called or attended the forum to obtain individual results; all 14 of these women also participated in the follow-up study. Once contacted for the follow-up study, an additional 16 participants (34%) who had not called to obtain results stated that they wanted individual results. Four commented that the method for distributing results was inconvenient, and that sending results via e-mail or a letter would have been preferable. Of the 31 people who participated in the follow up questionnaire, only one declined to receive individual results, noting, "I'm a little scared to see it... There isn't anything I can do to change things, so I'd rather not know."

The sixteen women who wanted individual data but had not called in prior to the follow-up study were given their results after they had finished the follow-up questionnaire. Their responses to the follow-up questionnaire were therefore not affected by individual results, and they are not included in the following section.

Of the 14 individuals who called or attended meetings to obtain results, five had stopped breastfeeding before results were made available, so individual results could not have affected the duration of breastfeeding. The women who were still breastfeeding when results were made available were asked, "Did learning about the PBDE levels in your milk affect how long you breastfed your baby?" Of the women who were still breastfeeding when results were made available, all nine continued to breastfeed at least until the time of the follow-up questionnaire. All reported that the results had not changed the duration that they planned to breastfeed. However, comments in response to follow-up probes suggested that there was potential for individual body burden information to affect their attitudes. For example, one participant noted, "If I had gotten very high results within a short time of testing, it might have affected me."

Two of the participants with results at the higher end of the range expressed concern about the potential for impacts on her baby's health. Both cited study literature and the "breastfeeding is best" message as factors in her comfort with breastfeeding as well as the availability of study staff to discuss results. One woman stated:

Learning about my own results made me nervous... made my husband nervous, but I continued because of the message that breastfeeding is best.

The other noted that the manner in which results were distributed helped her understand and feel comfortable with the results. She responded:

.... I was very affected by how the results were framed – talking about it in terms of averages, relations to animal studies, and the message that breastfeeding is best....

In addition, there were five participants who expressed relief that their results were in the lower end of the range, as one noted:

[low results] ... validated my decision to breastfeed...although with high results maybe I would have talked to my doctor about breastfeeding.

These responses demonstrate that there is potential for individual body burden results to create anxiety, and that the manner in which results are distributed must be considered as an important part of study design.

To gauge the effect that the overall study experience had had on decisions regarding breastfeeding, we asked all thirty-one participants of the follow-up study if they would breastfeed a second child if they had one. Thirty (97%) answered that they would breastfeed again; only one participant said that her decision would depend on her individual body burden results, but that she most likely would breastfeed again.

### Methodology for Distributing Results

One of the study questions focused on means by which individual results were distributed. While 30 (97%) of the follow-up study participants expressed an interest in personal results, only 14 (47%) called or attended meetings to obtain them. One of the barriers to accessing individual results was the requirement to call or attend a meeting. Many participants of the follow-up study mentioned how busy they were; six reported that they lost the first letter or forgot to call for results. While four participants of the follow-up study reported that they would have preferred to have results sent to them, two others stated that the manner in which results were distributed facilitated their understanding of the results and mitigated concerns.

### Impacts on Lifestyle

Recruiting materials and topics covered in the exposure questionnaire may have raised participant awareness about how household products and personal choices might affect one's health. We asked participants about changes they had made to their lifestyle because of the study. Fifteen participants (48%) felt that the study had no impact on their lifestyle or attitude. Eleven (35%) reported changes to the way they thought about their environment. Some of the responses demonstrated a heightened awareness of potential PBDE exposure within the home: "I thought about the foam things that I could do without," "Our couch got ripped, and I thought about PBDE...," "I became more aware of dairy products and consumption..."

Five participants (16%) cited tangible changes they had made to their lifestyle because of the study. Some attempted to reduce exposure to PBDE-treated products. For example, one participant reported, "we're being better about dust in the house." Other participants made changes that were related to overall health including choosing to eat more organic products and reducing sun exposure.

The biomonitoring study provided an opportunity for participants to learn about environmental health. This was seen as one of the benefits of the study by several participants, as articulated by one woman:

I found that there is a real lack of understanding about breastfeeding... it was hard to find information on how the mom's health and environment affects breastmilk.

## Discussion

### Attitudes Towards Breastfeeding

Overall, the findings of the follow-up questionnaire suggested that participation in the biomonitoring study, including reading recruitment materials, providing a breastmilk sample, and learning about individual body burden, did not negatively impact participants' attitudes towards breastfeeding or the duration of breastfeeding. However, some participants' responses suggested that there is potential for biomonitoring studies to raise concern about breastfeeding, and that aspects of our study design were able to mitigate negative impacts. Participants specifically mentioned the "breastfeeding is best" logo, the context in which results were provided, and the manner in which results were personally communicated. Our findings support the recommendations of the Technical Workshop outlined above in that breastfeeding support and clearly written, non-alarmist information appear to have mitigated potential negative impacts of the study. Future evaluation of biomonitoring participant experiences should focus on the details of study design more closely.

While the follow-up questionnaire focused on potential negative impacts, many participants considered their experience in the study to have had a positive impact, validating their decisions to breastfeed. Several of our participants had seen media coverage of previous biomonitoring studies and had concerns about the effect of bioaccumulation on breastmilk and infant health. Participants described our information as reassuring, informative, and accessible.

There was particular concern about participants from Lowell because the population historically has low breastfeeding rates. The experiences of this population could not be directly assessed due to lack of participation in the follow-up study; however, the director of the Lowell health center believed that the study had a positive impact by providing lactation information and support to patients and focusing staff attention on the need for more lactation education. Because of the differences between the Lowell group and other biomonitoring participants, it cannot be assumed that the findings of the follow-up study can be applied to participants from the Lowell group. Attitudes towards breastfeeding differ by class, race, and culture. It is important to assess the experiences of participants from different backgrounds in order to more fully understand the potential for impacts on different communities, particularly communities that are already affected by low breastfeeding rates.

It is difficult to compare the results of our follow-up study to the findings of the studies conducted following the Michigan PBB event. Hatcher [5] demonstrated the potential for individual biomonitoring information to negatively impact breastfeeding, but participants from the Hatcher study were recruited from a group of women who were sufficiently concerned about contamination to submit breastmilk for analysis. That study was also conducted shortly after the massive contamination episode, and was likely affected by the crisis atmosphere surrounding the event. Thomas participants were drawn from the general exposed population, and included women who gave birth up to twenty-five years following the accident. PBB was measured in blood, not breastmilk [7].

### Impacts of Individual Results on Breastfeeding

Many of the GB-PBDE participants who called to obtain results had questions about the interpretation of results and the potential for health impacts; however, all of the women who were breastfeeding at the time biomonitoring results were made available were still breastfeeding at the time of the follow-up study. All nine stated that the individual body burden results had not affected their decision about breastfeeding duration.

The potential for our study to impact breastfeeding duration may have been limited by the amount of time it took to collect samples and conduct analysis. Results were not available to participants until more than one year after the study was initiated, three to 15 months (average of nine months) after women had been recruited and breastfeeding had been initiated. Studies of breastfeeding practices have found that breastfeeding most often stops after 2–3 months [26]. The women who were still breastfeeding at the time our results were made available were already beyond the first months of breastfeeding and may have been more likely to continue breastfeeding regardless of body burden results. Providing individual results to women in their initial months of breastfeeding could potentially result in a greater negative impact on breastfeeding duration.

The time lag between sample collection and distribution of results may have affected how participants were impacted. The time-line of a study, including when report-back will be conducted, is an important consideration for study design, and is an example of the need for a standardized biomonitoring protocol.

### Distribution of Results

The GB-PBDE protocol allowed participants to choose whether they wanted to obtain individual results. We required that participants talk to study staff to obtain results. This procedure worked well for participants who had questions or concerns and appreciated immediate access to study staff. However, it reduced the number of participants who obtained results. Design of report-back protocol should consider both the effort required of participants to obtain results and the need to respond to participants' questions and concerns. This is difficult because the needs of individual participants vary widely. Not only is there a wide range in individual body burden results, but individuals also differ with respect to risk perception and risk tolerance [1]. Continued evaluation of result communication must be included in future biomonitoring studies in order to better understand the issue.

Distribution of individual results, particularly if conducted on an individual basis with counseling, has implications for research budgets. However, the cost considerations of providing individual results are outweighed by the need to treat participants with respect [11]. Standardization of biomonitoring report-back protocol which includes the need to distribute results on an individual basis will help researchers justify study timelines and budgets to IRB reviewers and funding agencies.

### Lifestyle and Attitudes

The GB-PBDE study was an opportunity to distribute information about biomonitoring and counterbalance misleading headlines about breastmilk. It was also an opportunity to raise awareness about chemicals in our everyday environment. The responses to questions about lifestyle and attitude changes demonstrated that the participants of the biomonitoring study were more aware of chemicals in consumer products as a result of the study.

### Study Limitations

There were several study design issues which may have affected the outcome of the follow-up study.

Participants may have been influenced by the fact that the study coordinator was a nursing mother. Perceived similarity between interviewers and subjects has been found to influence survey responses [27,28]. In both the biomonitoring and the follow-up study, participants may have been more willing to answer questions openly. The interviewer's status as a nursing mother may also have reduced concerns about breastfeeding or deterred participants from making negative statements about breastfeeding.

Results of the follow-up study may not be generalizable to more diverse populations. None of the biomonitoring participants from Lowell (n = 5) participated in the follow-up study. We do not have data on women who were eligible to participate but did not enroll. Nor do we have data on women who ultimately did not choose to breastfeed, including, perhaps, women who were negatively affected by the study recruitment literature. The study also does not address the potential impact of biomonitoring results on the general public, including women who might consider breastfeeding in the future.

Participants were contacted for the follow-up study at a time when their babies were between the ages of 4–18 months. Many of the participants were juggling the demands of motherhood and work; many mentioned how busy they were. Women were often rushed on the phone or interrupted by a crying baby, and the richness of the data was affected by the brevity of the interviews. Many of the women who did not initially call in to receive results mentioned time constraints or lack of organization as the reason they did not contact us. Participation in the follow-up study may have been similarly affected.

## Conclusion

The comments from our participants provided insight into the ways that participation in a breastmilk biomonitoring study can affect participants' attitudes towards breastfeeding. They also show that study design, including study literature and the method for report-back, can mitigate negative impacts. Standardization of biomonitoring studies is needed to fully protect participants' rights. The development of appropriate ethical standards requires input from many different sectors including researchers from varied disciplines such as risk communication, bioethics, and epidemiology; community groups and the lay public; clinicians and lactation consultants; and members of Institutional Review Boards. As the technology of biomonitoring advances, the discussion of the bioethics of biomonitoring must keep pace so that the Human Subject Research standards of respect for persons, beneficence, and justice can be upheld.

## Abbreviations

NRC: National Research Council; PBB: Polybrominated biphenyl; PBDE: Polybrominated diphenyl ether.

## Competing interests

The authors declare that they have no competing interests.

## Authors' contributions

NW was responsible for study design and implementation, data analysis, and drafting the manuscript. PB participated in data analysis and manuscript revision. AA reviewed study design and participated in manuscript revision. MM and TW participated in study design and contributed significantly to manuscript revision.

## Supplementary Material

Additional file 1 **The Value of Body-Burden Monitoring Using Breast Milk.** Breast milk biomonitoring fact sheet distributed to potential biomonitoring participants. Click here for file

Additional file 2 **Out of Harm's Way: Preventing Toxic Threats to Child Development – Why Breast-Feeding is Still Best for Baby.** Educational information distributed to potential biomonitoring participants. Click here for file

Additional file 3 **The Greater Boston PBDE Body Burden Project.** Fact sheet on polybrominated diphenyl ethers (PBDEs) distributed to potential biomonitoring participants. Click here for file

Additional file 4 **Follow-up Questionnaire.** Questionnaire used in the follow-up study to collect information on participants' experiences in the biomonitoring study. Click here for file
