# Supplementary material for: Participant experiences in a breastmilk biomonitoring study: A qualitative assessment
Source: Environ Health. 2009 Feb 18;8:4. doi: 10.1186/1476-069X-8-4 (PMC2649062; doi:10.1186/1476-069X-8-4)
Supplement: Additional file 1 — The Value of Body-Burden Monitoring Using Breast Milk. Breast milk biomonitoring fact sheet distributed to potential biomonitoring participants. [file 1476-069X-8-4-S1.doc]

**Greater Boston PBDE Body Burden Project**

**The Value of Body-Burden Monitoring Using Breast Milk**

***Body burden studies are a way to track environmental exposure to chemicals***

We are exposed to a variety of chemicals through the air we breathe, the food we eat, the water we drink, and the products we use. While exposure to some chemicals can be measured through environmental monitoring, exposure to other chemicals is more accurately measured in body tissue. Body burden monitoring can help estimate an individual’s exposure to certain chemicals and provide time and geographic trend information, showing us how chemical exposure may vary over time and by community.

***How is body burden data used?***

Body burden data can be helpful in the identification of disease risk factors and important routes of exposure. The data also can be used to promote proactive environmental regulations. In Sweden, data which showed increasing rates of PBDE concentrations in breast milk over time led to regulations limiting the use of these compounds by industry. Currently there are no on-going breast milk monitoring programs in the United States.

***Why is breast milk used for body burden monitoring?***

Some of the most persistent chemicals such as PCBs, dioxins, and PBDEs are lipophilic (fat loving). Fat is present in blood and fat tissue as well as in breast milk. Breast milk monitoring is a relatively non-invasive means of measuring the level of chemicals present in a nursing mother. The presence of chemicals in breast milk close to the time of delivery gives us an accurate measure of the mother’s body. Body burden data also can provide an estimate of the chemical concentrations to which the infant was exposed during gestation; during pregnancy, chemicals in the mother’s body can cross the placenta and enter the body of a developing fetus.

***Breast feeding is still the best option for your baby***

Breast milk is still the best food that you can give your baby. Despite the presence of some chemicals in breast milk, studies show that breastfed babies are healthier. In prior studies, breast feeding has been shown to reduce the impacts of *in utero* exposure to chemicals. By promoting breast milk body monitoring programs and proactively working to identify chemical contaminants in breast milk, we hope to maintain breast milk as the precious resource that it is, and to promote healthy breast feeding practices.

For more information on body burden monitoring, please visit these websites:

Environmental Working Group: <http://www.ewg.org/reports/bodyburden/>

Centers for Disease Control Body Burden Study: <http://www.cdc.gov/exposurereport/default.htm>

Physicians for Social Responsibility: [http://www.psr.org](http://www.psr.org/) – click on Environment and Health and “Bearing the Burden”

Additional references on body burden monitoring:

Hooper, K. and She, J. 2003. Lessons from the polybrominated diphenyl ethers (PBDEs): Precautionary principle, primary prevention, and the value of community-based body-burden monitoring using breast milk. Environmental Health Perspectives 111(1): 109-114.

Landrigan, P.J., Sonawane, B., Mattison, D., McCally, M., and Garg, A. 2002. Chemical contaminants in breast milk and their impacts on children’s health: An overview. Environmental Health Perspectives 110(6): 313-315.E

For more information on this study, please contact:

Nerissa Wu, MPH, Boston University School of Public Health

(617) 638-4620

[nerissw@bu.edu](mailto:nerissw@bu.edu)
