# Supplementary material for: Participant experiences in a breastmilk biomonitoring study: A qualitative assessment
Source: Environ Health. 2009 Feb 18;8:4. doi: 10.1186/1476-069X-8-4 (PMC2649062; doi:10.1186/1476-069X-8-4)
Supplement: Additional file 2 — Out of Harm's Way: Preventing Toxic Threats to Child Development – Why Breast-Feeding is Still Best for Baby. Educational information distributed to potential biomonitoring participants. [file 1476-069X-8-4-S2.pdf]

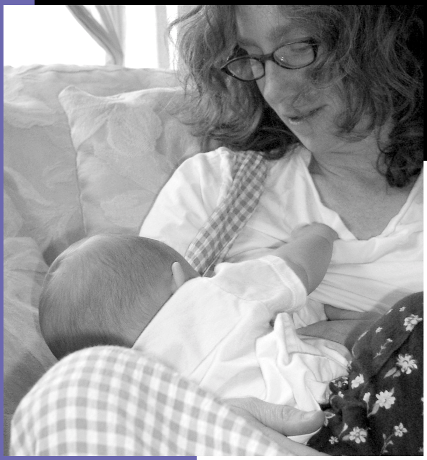

Out of Harm's Way:

Preventing Toxic Threats to Child Development

Why Breast-Feeding is Still Best for Baby

This fact sheet provides women, their families, and their health care providers with a summary of current scientific evidence that shows that even in a polluted world, breast is best.

Each of us wants to help our babies be as healthy as possible. One way to do that is to breast-feed your baby. Doctors agree that breast milk is the best food for your child.

You may have heard that pollution can make its way into the breast milk of nursing mothers. Does that mean that it is better to feed formula to your baby than to breast-feed?

The answer is NO! Breast milk is still by far the best food for your baby.

Summary

- Breast-feeding is still the best way a mother can feed her baby.
- Breast-feeding is healthier than formula bottle-feeding for both babies and mothers.
- Both baby formula and breast milk may contain chemicals that may be harmful. However, the good things about breast-feeding outweigh any risks from pollution that may have made its way into breast milk.
- Mothers and mothers-to-be can make their breast milk safer by eating less animal fat. Animal fat is found in foods like cheese, meat and fish. It is even better if the woman starts eating less animal fat a long time before she gets pregnant.
- Not making or using harmful chemicals in our environment is the best way to protect mothers and babies from the bad effects of harmful chemicals.

Why Breast-Feeding is Still Best for Baby

With all the concern about chemicals in breast milk, should women breast-feed their babies?

Yes. While we do not like to have chemicals in breast milk, studies do not find any increase in health risks for your baby from typical levels of chemicals in breast milk. However, studies do show that babies fed bottle formula may get sick more often than babies who are breast-fed. Breast-feeding provides special benefits for both babies and mothers. The American Academy of Pediatrics says that breast milk is the best food of all for your baby.

The American Academy of Pediatrics says that breast milk is the best food of all for your baby.

What are some of the good things about breast-feeding?

Breast-feeding reduces the chance that your baby will get many different diseases. Breast-fed babies are less likely to get pneumonia, diarrhea, ear infections, and some other infections caused by germs. And, if your baby does get sick, the sickness will probably not be as bad if your baby is breast-fed.

Breast-feeding also helps the baby's brain grow properly, and reduces the chance that s/he will develop asthma, cancer, diabetes, or become overweight. Breast-feeding for longer periods of time (pediatricians suggest at least one year) seems to be even better. Breast-feeding also helps mother and baby feel closer and more loving.

Breast-feeding is good for mothers, too.

Breast-feeding is also good for the health of mothers. These good things include less bleeding after the birth, a faster return to pre-pregnancy weight, better bone strength, fewer hip fractures in later life, and less chance of getting ovarian and breast cancers when she is young (still getting her period).

How do the chemicals in breast milk affect a baby's health?

Chemical exposures to a baby still growing in the mother's womb are known to be bad, but common exposures through breast-feeding have not been shown to cause harm to the baby. This may be because the baby is stronger after s/he is born vs. still in the womb, or because breast milk helps protect the baby in other ways.

While chemicals in breast milk do not seem to cause harm, breast milk with high amounts of chemicals can reduce some of the good things about breast-feeding. Large amounts of chemicals called PCBs, for example, seem to reduce the resistance to infection that comes with breast-feeding. As a result, babies who take breast milk with very high PCB levels may get sick as much as formula-fed babies.

Baby formula can contain chemicals, too.

In addition to increasing risks for some health problems, formulas also may contain harmful chemicals like pesticides (which are used to kill insects). Formula is also high in a metal called "manganese" (10-50 times more than breast milk). This may be bad because too much manganese may affect behavior and attention in children. Manganese levels are highest in supplemented and soy-based formulas.

Women can make their breast milk even safer.

A low-fat diet is best from childhood through adulthood. Because many chemicals collect in animal fat, it is a good idea for women to eat less animal fat beginning after two years of age. This decreases the build-up of harmful chemicals in the body. That in turn reduces the amount of chemicals in breast milk. Even more important, eating less animal fat decreases the unborn baby's exposure to chemicals in mother's body that may be harmful.

To reduce animal fat, eat fewer animal products in general, and choose nonfat or low-fat animal foods such as skimmed milk and lean poultry, beef, and fish. It is especially helpful to avoid processed foods made from ground meat and animal parts such as sausage, bologna, hot dogs, and canned, ground lunchmeats. Eating more fruits, vegetables, beans, grains, and low-fat or nonfat animal products gives you good nutrition and reduces the level of toxic chemicals in the body.

Reduce or End the Production of Harmful Chemicals

Many toxic chemicals that make their way into food last for years in the environment and build up in the body. Other harmful chemicals in products and the environment do not last as long, but are also dangerous because they can pass easily from a mother's body to her unborn baby or nursing infant.

As long as our society produces and uses chemicals like these, we cause pollution in the environment, our food, and our bodies. The best way to keep breast-feeding safe, protect the health of babies in the womb, and offer children the healthy start they deserve is to reduce or stop the creation and use of harmful chemicals.

References to this companion fact sheet can be found in the report *In Harm's Way: Toxic Threats to Child Development,* issued by Greater Boston Physicians for Social Responsibility (GBPSR) in May, 2000. It was revised in the Fall of 2002 by GBPSR, San Francisco PSR, the Institute for Agriculture and Trade Policy, and Clean Water Fund, California. The 140-page report can be viewed, downloaded, or ordered at http://www.igc.org/psr/. It is part of a series for the project *In Harm's Way Training Materials for Health Professionals.*

For more information on this and other fact sheets in the series, contact: Greater Boston Physicians for Social Responsibility, 11 Garden St., Cambridge, MA 02138. 617-497-7440. psrmabo@igc.org. In California, you can contact San Francisco Bay Area Physicians for Social Responsibility, 2288 Fulton Street, Suite 307, Berkeley, CA 94704. 510-845-8395. info@sfbaypsr.org. Or, Clean Water Fund, 814 Mission Street, Suite 602, San Francisco, CA 94103 (415) 369-9160. cwasf@cleanwater.org.

This project has been supported by the Jessie B. Cox Charitable Trust, The Richard and Rhoda Goldman Fund, the Jewish Healthcare Foundation, the W. Alton Jones Foundation, the Mitchell Kapor Foundation, The John Merck Fund, the Alida R. Messinger Charitable Lead Trust, The San Francisco Foundation, and the Environmental Protection Agency, Office of Children's Health Protection.

♻ Printed on process chlorine free paper, 30% post-consumer waste, with soy-based inks.
Graphics: Stephen Burdick Design; Photo: Julie L. Silas
