# Supplementary material for: Participant experiences in a breastmilk biomonitoring study: A qualitative assessment
Source: Environ Health. 2009 Feb 18;8:4. doi: 10.1186/1476-069X-8-4 (PMC2649062; doi:10.1186/1476-069X-8-4)
Supplement: Additional file 3 — The Greater Boston PBDE Body Burden Project. Fact sheet on polybrominated diphenyl ethers (PBDEs) distributed to potential biomonitoring participants. [file 1476-069X-8-4-S3.doc]

**The Greater Boston PBDE Body Burden Project**

**Boston University School of Public Health**

**Lowell Center for Sustainable Production at UMass Lowell**

**What are PBDEs**?

Polybrominated diphenyl ethers, or PBDEs, are a group of compounds used as flame retardants. They are added to plastics, foams, and textiles in consumer products including furniture, mattresses, pillows, televisions, and computers. Recent studies discovered that the concentrations of PBDEs in people have been increasing over the last few decades. The very limited data from the United States suggest that levels are many times greater than in Europe, but the reason for this difference is unknown. The main routes of exposure have not been established, although both food and direct exposure via consumer products are suspected. PBDEs are persistent and accumulate in body fat. Human exposure is currently lower than the levels at which known health effects, e.g., toxicity to the nervous system, are seen in laboratory animals. But since PBDEs are widely used in the U.S., the concentrations in the environment and in human tissue are likely to increase. Little is known about the effects of exposure during key periods of development, particularly exposure in the womb.

**What is the goal of the Greater Boston PBDE Body Burden Project?**

Our ultimate goal is to determine the levels of PBDEs in human tissues in the Greater Boston Area and to identify key exposure pathways. This is an important step towards designing strategies for preventing accumulation of these compounds in people. The first phase of our study will investigate body burdens in the Merrimack Valley and Greater Boston area by measuring PBDEs in human milk. We are also conducting environmental sampling inside homes to determine the concentration of PBDEs in house dust.

**Why measure body burdens using human milk?**

PBDEs accumulate primarily in fat, but their concentrations are still quite low. Human milk (breast milk) is usually about 3-4% fat and provides a convenient, relatively non-invasive way to estimate the concentrations in people's fat. The World Health Organization (WHO) has measured other fat-soluble compounds (such as polychlorinated biphenyls, or PCBs) in human milk for some time. ***WHO and many other groups, such as the American Academy of Pediatricians, strongly recommend human milk as the best food for infants. Our group agrees; we want to both promote breastfeeding and learn more about the levels of PBDEs in people.***

**Who is eligible to participate?**

In order to ensure comparability with studies in other areas, this study will be modeled after WHO criteria for selecting participants. First-time mothers over the age of eighteen who will have lived in the Greater Boston area for three or more years at the time of delivery are eligible to participate. Mothers and infants should be healthy; pregnancies should be singlet and normal. Mothers must be planning to breast feed for the first two months after delivery so that human milk samples can be collected from two to eight weeks after delivery.

**How will the study work?**

We are recruiting women during the late stages of pregnancy. We will conduct a short interview to collect data on diet, as well as potential exposure to consumer goods which may contain PBDEs. After the interview, study participants will be given sample collection bottles and instructions on how to take a sample. We are asking each participant to provide one sample of about 50 ml (approximately 1/4 cup) during the first two months of breast feeding. Ideally, samples will be hand expressed, but manual or electric pumps can also be used. We hope to make participation as convenient and non-invasive as possible. Sample jars will be provided and picked up, manual breast pumps will be available to participants as needed, and a small stipend will be given in appreciation for participation. Results of the study as well as results for individual analysis will be made available to participants. ***A physician from Greater Boston Physicians for Social Responsibility as well as study staff will be available to answer any questions you may have about the purpose of the study or about your individual results.***

**What part can you play?**

If you are eligible to participate in this study and would like to enroll, or if you are interested in obtaining more information, please contact us at:

Nerissa Wu, MPH, Boston University School of Public Health

(617) 638-4620

[nerissw@bu.edu](mailto:nerissw@bu.edu)

***Remember: Breast feeding is best!***
