# Supplementary material for: Participant experiences in a breastmilk biomonitoring study: A qualitative assessment
Source: Environ Health. 2009 Feb 18;8:4. doi: 10.1186/1476-069X-8-4 (PMC2649062; doi:10.1186/1476-069X-8-4)
Supplement: Additional file 4 — Follow-up Questionnaire. Questionnaire used in the follow-up study to collect information on participants' experiences in the biomonitoring study. [file 1476-069X-8-4-S4.doc]

**Follow-Up Questionnaire**

1. How did you first hear about the Greater Boston PBDE Body Burden Project?

2. At what stage in your pregnancy did you first hear about the Project?

3. Before you heard about the Project, what were your plans for breastfeeding? Were you:

_____ Already breastfeeding or definitely planning to breastfeed

_____ Very likely to breastfeed

_____ Considering breastfeeding, but not sure.

4. Did the information given to you about the study change how you felt about breastfeeding? If yes, please describe.

5. For how long did you breastfeed your baby?

_____ 2 months or less

_____ 2-6 months

_____ 6-12 months

_____ >12 months

6. Was the duration that you breastfed your baby affected by the information you were given as part of the Project? Please describe.

7. Can you describe any changes to your lifestyle that you have made because of your participation in the study?

8. Did you choose to receive the results of your personal milk analysis? (If no, skip to Question 12

9. Did learning about the PBDE levels in your milk affect how you felt about breastfeeding?

10. Did learning about the PBDE levels in your milk affect how long you breastfed your baby?

11. If you have another baby, do you think you will choose to breastfeed?

12. Was there any part of how the study was administered (how initial information was provided, how you were contacted, how study results were distributed) that you would have liked to see done differently?

13. Have you heard recent news reports about PBDEs, and if so, has that affected your response to this research?

14. Have you talked with other new mothers who participated in the study? If so, what thoughts and ideas did you share?

15. Do you have any more questions about the study or your personal results that I could answer at this time?
